# Single-cell RNA-seq denoising using a deep count autoencoder

**DOI:** 10.1038/s41467-018-07931-2

**Published:** 2019-01-23

**Authors:** Gökcen Eraslan, Lukas M. Simon, Maria Mircea, Nikola S. Mueller, Fabian J. Theis

**Affiliations:** 1 Institute of Computational Biology, Helmholtz Zentrum München, Neuherberg, Germany; 2 TUM School of Life Sciences Weihenstephan, Technische Universität München, Freising, Germany; 3 Department of Mathematics, Technische Universität München, Garching, Germany

## Abstract

Single-cell RNA sequencing (scRNA-seq) has enabled researchers to study gene expression at a cellular resolution. However, noise due to amplification and dropout may obstruct analyses, so scalable denoising methods for increasingly large but sparse scRNA-seq data are needed. We propose a deep count autoencoder network (DCA) to denoise scRNA-seq datasets. DCA takes the count distribution, overdispersion and sparsity of the data into account using a negative binomial noise model with or without zero-inflation, and nonlinear gene-gene dependencies are captured. Our method scales linearly with the number of cells and can, therefore, be applied to datasets of millions of cells. We demonstrate that DCA denoising improves a diverse set of typical scRNA-seq data analyses using simulated and real datasets. DCA outperforms existing methods for data imputation in quality and speed, enhancing biological discovery.

## Abstract (web summary)

Single-cell RNA sequencing is a powerful method to study gene expression, but noise in the data can obstruct analysis. Here the authors develop a denoising method based on a deep count autoencoder network that scales linearly with the number of cells, and therefore is compatible with large data sets.

## Introduction

Advances in single-cell transcriptomics have enabled researchers to discover novel celltypes^1,2^, study complex differentiation and developmental trajectories^3–5^ and improve understanding of human disease^1,2,6^.

Despite improvements in measuring technologies, various technical factors, including amplification bias, cell cycle effects^7^, library size differences^8^ and especially low RNA capture rate^9^ lead to substantial noise in scRNA-seq experiments. Recent droplet-based scRNA-seq technologies can profile up to millions of cells in a single experiment^10–12^. These technologies are particularly sparse due to relatively shallow sequencing^13^. Overall, these technical factors introduce substantial noise, which may corrupt the underlying biological signal and obstruct analysis^14^.

The low RNA capture rate leads to failure of detection of an expressed gene resulting in a “false” zero count observation, defined as dropout event. It is important to note the distinction between “false” and “true” zero counts. True zero counts represent the lack of expression of a gene in a specific celltype, thus true celltype-specific expression. Therefore, not all zeros in scRNA-seq data can be considered missing values. In statistics, missing data values are typically imputed. In this process missing values are substituted for values either randomly or by adapting to the data structure, to improve statistical inference or modeling^15^. Due to the non-trivial distinction between true and false zero counts, classical imputation methods with defined missing values may not be suitable for scRNA-seq data.

**Fig. 1:**
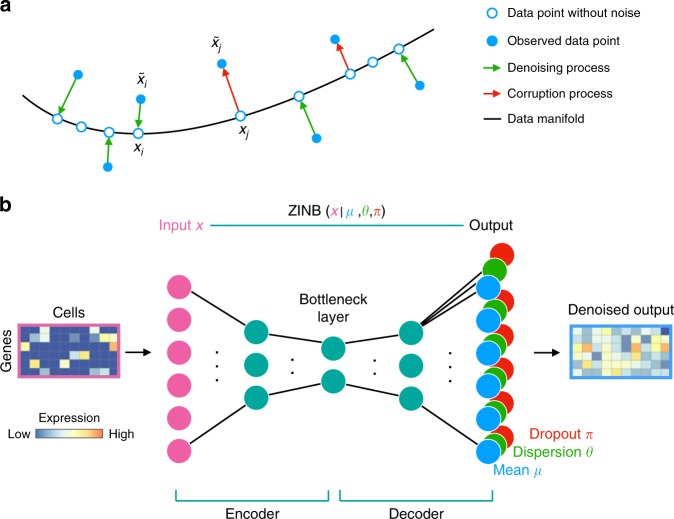
DCA denoises scRNA-seq data by learning the underlying true zero-noise data manifold using an autoencoder framework. **a** Depicts a schematic of the denoising process adapted from Goodfellow et al.^24^. Red arrows illustrate how a corruption process, i.e. measurement noise including dropout events, moves data points *x*_*j*_ away from the data manifold (black line). The autoencoder is trained to denoise the data by mapping measurement-corrupted data points ${\tilde{x}}_{i}$ back onto the data manifold (green arrows). Filled blue dots represent corrupted data points. Empty blue points represent the data points without noise. **b** Shows the autoencoder with a ZINB loss function. Input is the original count matrix (pink rectangle; gene by cells matrix, with dark blue indicating zero counts) with six genes (pink nodes) for illustration purposes. The blue nodes depict the mean of the negative binomial distribution which is the main output of the method representing denoised data, whereas the green and red nodes represent the other two parameters of the ZINB distribution, namely dispersion and dropout. Note that output nodes for mean, dispersion and dropout also consist of six genes which match six input genes. The matrix highlighted in blue shows the mean value for all cells which denotes the denoised expression. and the mean matrix of the negative binomial component represents the denoised output (blue rectangle). Input counts, mean, dispersion and dropout probabilities are denoted as *x*, *μ*, *θ* and *π*, respectively

The concept of denoising is commonly used to delineate signal from noise in imaging^16^. Denoising enhances image quality by suppressing or removing noise in raw images. We assume that the data originates from a noiseless data manifold, representing the underlying biological processes and/or cellular states^17^. However, measurement techniques like imaging or sequencing generate a corrupted representation of this manifold (Fig. 1a).

A number of scRNA-seq specific imputation or denoising methods exist^18–22^. These approaches rely on using the correlation structure of single-cell gene expression data to infer “corrected” gene expression values by leveraging information on similarities between cells and/or genes. For example, current approaches for scRNA-seq specific imputation include scImpute^22^, which defines likely dropout values using a mixture model and subsequently substitutes only the likely dropout values. MAGIC^20^ and SAVER^21^, on the other hand, denoise single-cell gene expression data and generate a denoised output for each gene and cell entry. However, these methods may fail to account for non-linearity or the count structure in the data. Furthermore, with the increasing size of scRNA-seq datasets^13^, methods need to scale to up to millions of cells and existing denoising methods are unable to process datasets of this magnitude.

An autoencoder is an artificial neural network which learns an efficient compression of data in an unsupervised fashion by minimizing the error between the compressed and subsequently reconstructed data set versus the original one. Generalizing linear approaches such as principal component analysis, it is commonly used for dimension reduction^23^ (see Methods for the detailed description of autoencoders). Since the compression forces the autoencoder to learn only the essential latent features, the reconstruction ignores non-essential sources of variation such as random noise^24^ (Fig. 1a). A number of recent studies describe applications of autoencoders in molecular biology^25–29^.

To solve denoising and imputation tasks in scRNA-seq data in one step, we extend the typical autoencoder approach and adapt it towards noise models applicable to sparse count data. To that end, we developed a deep learning based autoencoder with specialized loss functions targeted towards scRNA-seq data, the so-called “deep count autoencoder” (DCA). The trick is to define the reconstruction error as the likelihood of the distribution of the noise model instead of reconstructing the input data itself (Fig. 1b). During training, DCA learns gene-specific distribution parameters by minimizing the reconstruction error in an unsupervised manner. Due to the compression, DCA shares information across features, and thereby accounts for gene-gene dependencies. The deep learning framework (by default three hidden layers with 64, 32, 64 neurons) of DCA enables the capturing of the complexity and non-linearity in scRNA-seq data. Thirdly, the autoencoder framework is highly scalable and DCA can be applied to data sets of up to millions of cells. To increase speed even further DCA is parallelizable via graphical processing units (GPU).

One of the main advantages of DCA is that the user only needs to specify the noise model. Existing scRNA-seq methods are based on various distributional assumptions, including zero-inflated negative binomial models^30,31^. However, Chen et al.^32^ proposed that zero-inflation is less likely in unique molecular identifier (UMI) based compared to read based scRNA-seq technologies. Therefore, to provide maximal flexibility, DCA implements a selection of scRNA-seq specific noise models including negative binomial distribution with (ZINB) and without zero-inflation (NB). For example, using the ZINB noise model, DCA learns gene-specific parameters mean, dispersion and dropout probability based on the input gene expression data. The inferred mean parameter of the distribution represents the denoised reconstruction and the output of DCA (Fig. 1b).

We extensively evaluate our approach with competing methods using simulated and real datasets. Altogether, we demonstrate that DCA shows high scalability and DCA denoising enhances biological discovery. The approach is implemented in Python and as a command line tool, publicly available at https://github.com/theislab/dca. Alternatively, Scanpy^33^ users can directly use the “dca” method in the preprocessing package[https://scanpy.readthedocs.io/en/latest/api/index.html#imputation].

## Results

### Count noise model is necessary to denoise scRNA-seq data

As a proof of principle and to explore the properties of our approach, we applied DCA to simulated scRNA-seq data generated using Splatter^34^. Both count data with and without dropout are available, which allows quantification of denoising using ground truth. We simulated two data sets with 200 genes and (1) two celltypes (2000 cells in total) and (2) six celltypes (2000 cells in total). For the two and six celltype simulations 63 and 35% of data values were set to zero, respectively. Dropout simulation probabilities are conditioned on mean gene expression, such that lowly expressed genes have a higher likelihood of dropout compared to highly expressed genes^34^.

To guide the user’s choice of the appropriate noise model, we propose to examine the relationship between the gene-wise mean and empirical dropout rate calculated for cells from the same cluster or cell type. By conducting a likelihood ratio test between the NB and ZINB fits the user can determine whether zero-inflation is present and which distribution to select for the DCA noise model parameter. For the simulation data, the ZINB distribution showed higher likelihood compared to NB distribution (Supplementary Fig. 1A). Therefore, we used the ZINB noise model for DCA denoising.

**Fig. 2:**
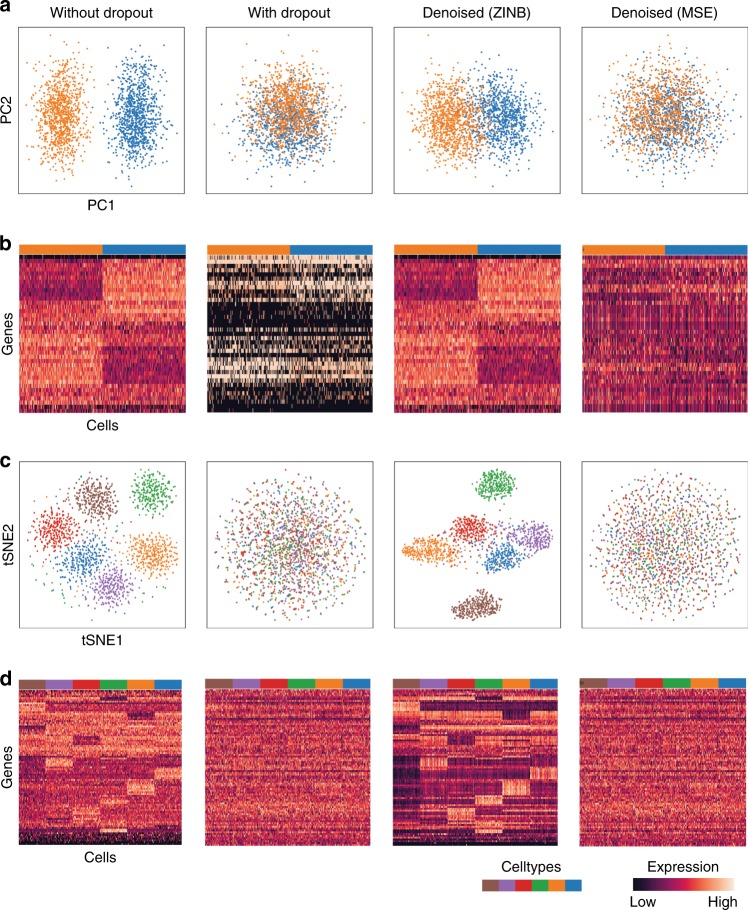
Count-based loss function is necessary to identify celltypes in simulated data with high levels of dropout noise. **a** depicts plots of principal components 1 and 2 derived from simulated data without dropout, with dropout, with dropout denoised using DCA and MSE based autoencoder from left to right. Cells are colored by celltype. **b** shows heatmaps of the underlying gene expression data. **c** illustrates tSNE visualization of simulated scRNA-seq data with six cell types. Cells are colored by celltype. **d** shows heatmaps of the underlying gene expression data

In our simulation results dropout adds substantial noise, obscuring celltype identities. Expectedly, after denoising using DCA the original celltypes can be recovered (Fig. 2a, b). To test whether a count-based loss function is necessary, we compared DCA to a typical autoencoder with a mean squared error (MSE) loss function using log-transformed count data. The MSE based autoencoder was unable to recover the celltypes, indicating that the specialized count loss function is necessary for scRNA-seq data. Confirmatory results were observed in the more complex six group simulation (Fig. 2c, d, Supplementary Fig. 2A & B).

One advantage of simulated data is the ability to perform a large variety of evaluations, including the assessment of potential overimputation. Overimputation in denoising methods manifests itself by introducing spurious correlations, falsely generating correlations between genes. The simulations contain two sets of genes which 1) show differential expression (DE) between celltypes, i.e. marker genes, and 2) which show no DE, i.e. housekeeper genes. Spurious correlations could falsely change housekeeper genes into marker genes. The DE genes drive the PCA, whereas the non-DE genes are expected to show no effect on the PCA. Therefore, we performed PCA on the denoised data using the subset of only non-DE genes (housekeepers) as input. After DCA denoising, celltype identities were not recovered, indicating that the denoising process did not introduce spurious correlations and is robust to overimputing (Supplementary Fig. 2C & D).

To test if DCA is capable of distinguishing true “celltype specific” from false “dropout” zero counts, we denoised the two group simulation data using hyperparameter settings that regularize for model complexity (see “Methods” section for details). Since the dropout effect is added on top of the simulation, the ground truth for each zero count is known. After DCA denoising we investigated the distribution of the inferred dropout probabilities as captured in the *π* parameter (Supplementary Fig. 2E, Fig. 1b). The inferred dropout probability for “dropout” zeros was much higher compared to “celltype specific” zeros, demonstrating the ability of DCA to discern zero counts (Supplementary Fig. 2F).

### DCA captures cell population structure in real data

**Fig. 3:**
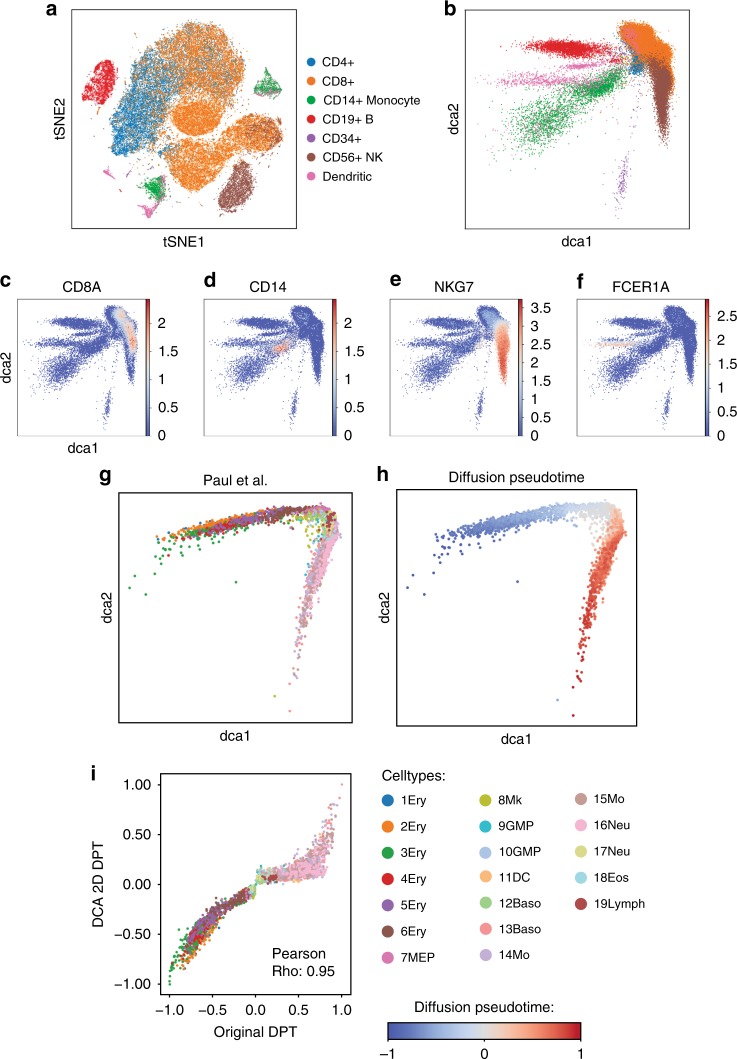
DCA captures population structure in 68,579 peripheral blood mononuclear cells. **a** shows the tSNE visualization reproduced from Zheng et al.^12^. **b** illustrates the activations from the two-dimensional bottleneck layer of the DCA. Colors represent celltype assignment from Zheng et al.^12^, where CD4 + and CD8 + cells are combined into coarse groups. Silhouette coefficients are −0.01 and 0.07 for tSNE and DCA visualizations. **c**–**f** show two-dimensional bottleneck layer colored by the log-transformed expression of celltype marker genes CD8A (CD8 + T cells), CD14 (CD14 + Monocytes), NKG7 (CD56 + natural killer cells) and FCER1A (dendritic cells), respectively. DCA derived manifold robustly reconstructs continuous differentiation phenotype. **g**, **h** illustrate the activations from the two-dimensional bottleneck layer of DCA colored by celltype assignment from Paul et al. (**g**) and diffusion pseudotime (**h**), respectively. **i** shows the DPT as calculated using the standard DPT workflow and the two-dimensional bottleneck layer coordinates on the *X* and *Y* axis, respectively. Cells are colored by celltype assignment from Paul et al.^35^. Abbreviations Ery, Mk, DC, Baso, Mo, Neu, Eos, Lymph correspond to erythrocytes, megakaryocytes, dendritic cells, basophils, monocytes, neutrophils, eosinophils and lymphoid cells, respectively

Complex scRNA-seq datasets, such as those generated from a whole tissue, may show large cellular heterogeneity. Therefore, denoising methods must be able to capture the cell population structure and use cell population specific parameters for the denoising process. To test whether DCA was able to capture cell population structure in real data we denoised scRNA-seq data of 68,579 peripheral blood mononuclear cells^12^ and 1,000 highly variable genes (92% zeros) (Fig. 3a). NB and ZINB model fits showed comparable goodness-of-fit based on likelihood ratio test (Supplementary Fig. 1B). In this situation, we advise using the NB noise model, since it is less complex and hence is easier to fit. For this analysis only, we restricted the autoencoder bottleneck layer to two neurons and visualized the activations of these two neurons for each cell in a two-dimensional scatter plot (Fig. 3b). When overlaying the original celltype information^12^, celltype-specific clustering was observed. Furthermore, known celltype marker genes showed cluster-specific expression in the two-dimensional bottleneck visualization (Fig. 3c–f), indicating that DCA captures the data manifold in real data and consequently cell population structure.

To investigate whether DCA is also able to capture a continuous phenotype, we performed analogous analysis using scRNA-seq data from continuous blood differentiation^35^. When visualizing the two-neuron bottleneck layer, the two differentiation trajectories towards megakaryocyte–erythroid progenitors (MEP) and granulocyte-macrophage progenitors (GMP) were revealed (Fig. 3g). Additionally, diffusion pseudotime (DPT) was calculated based on the 1) two-neuron bottleneck coordinates (Fig. 3h) and 2) alternatively on the gene expression PCA coordinates as is suggested in the standard DPT workflow^3^. We observed a strong correlation between the pseudotime values derived from the two manifolds, indicating that the DCA bottleneck layer can capture a continuous phenotype (Fig. 3i). Overall, these results demonstrate that DCA captures meaningful biological information. Therefore, DCA can derive cell population specific denoising parameters in an unsupervised fashion. Furthermore, the low-dimensional DCA representation can be used for downstream analyses, such as pseudotemporal ordering.

### Denoising recovers time-course patterns upon noise induction

**Fig. 4:**
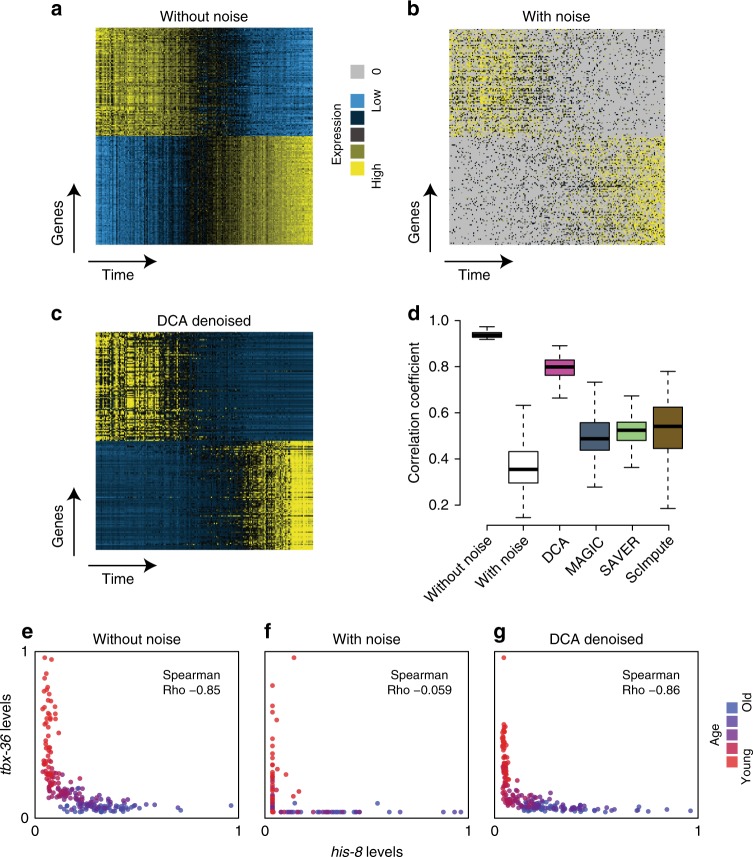
DCA recovers gene expression trajectories in *C*. *elegans* time course experiments with simulated dropout. Heatmaps show the top 100 genes with positive and negative association with time course using expression data without noise (**a**), with noise (**b**) and after DCA denoising (**c**). Yellow and blue colors represent relative high and low expression levels, respectively. Zero values are colored grey. Distribution of Pearson correlation coefficients across the 500 most highly correlated genes before noise addition for the various expression matrices are depicted in **d**. The box represents the interquartile range, the horizontal line in the box is the median, and the whiskers represent 1.5 times the interquartile range. Panels **e**–**g** illustrate gene expression trajectory for exemplary anti-correlated gene pair *tbx-36* and *his-8* over time for data without, with noise and after denoising using DCA

Next, we evaluated DCA by performing a systematic comparison with MAGIC^20^, SAVER^21^ and scImpute^22^ (Supplementary Table 1). We adapted the evaluation approach from van Dijk et al.^20^ and analyzed real bulk transcriptomics data from a developmental C. elegans time course experiment^36^ after simulating single-cell specific noise. Bulk contains less noise than single-cell transcriptomics data^37^ and can thus aid the evaluation of single-cell denoising methods by providing a good ground truth model. Gene expression was measured from 206 developmentally synchronized young adults over a twelve-hour period (Fig. 4a). Single-cell specific noise was added in silico by gene-wise subtracting values drawn from the exponential distribution such that 80% of values were zeros^20^ (Fig. 4b). DCA denoising recovered original time course gene expression pattern while removing single-cell specific noise (Fig. 4c). To systematically evaluate the four methods, we tested which method would best recover the top 500 genes most strongly associated with development in the original data without noise. DCA demonstrated the strongest recovery of these genes, outperforming the other methods (Fig. 4d). Gene-level expression without, with noise and after DCA denoising for key developmental genes *tbx-36* and *his-8* is depicted in Fig. 4e, f, g, respectively. Expression data derived from denoising using MAGIC, SAVER and scImpute for these two genes is displayed in Supplementary Fig. 4. *tbx-36* and *his-8* represent transcription factor and histone gene classes, respectively, which are known to show opposing expression patterns during C.elegans development^38^.

### Denoising improves differential expression analysis

**Fig. 5:**
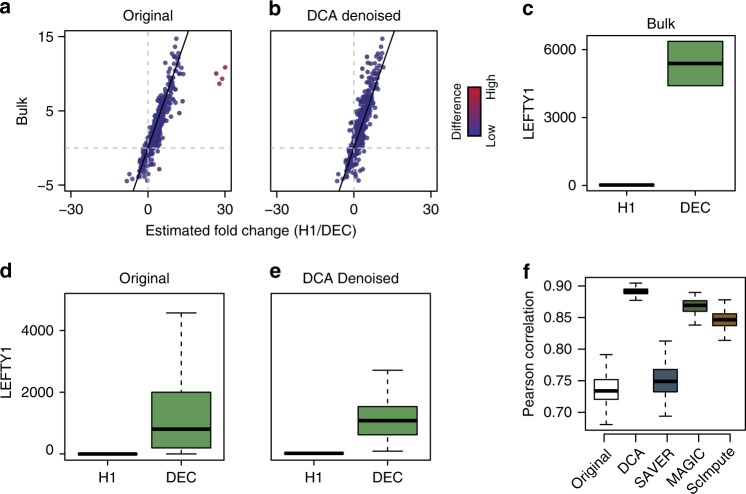
DCA increases correspondence between single-cell and bulk differential expression analysis. Scatterplots depict the estimated log fold changes for each gene derived from differential expression analysis using bulk and original scRNA-seq count matrix (**a**), DCA denoised count matrix (**b**). Grey horizontal and vertical lines indicate zero log fold change. Black line indicates identity line. Points are colored by the absolute difference between log fold changes from bulk and single-cell data with red colors indicating relative high differences. **c**–**e** depict differential expression of an exemplary gene *LEFTY1* between H1 and DEC for the bulk, original and DCA denoised data, respectively. **f** illustrates boxplots of the distribution of Pearson correlation coefficients from bootstrapping differential expression analysis using 20 randomly selected cells from the H1 and DEC populations for all denoising methods

Motivated by the scRNA-seq denoising evaluation metrics proposed by Li et al.^22^, we compared differential expression analysis results between bulk and scRNA-seq data from the same experiment. Chu et al^39^. generated bulk and scRNA-seq data from H1 human embryonic stem cells (H1) differentiated into definitive endoderm cells (DEC). The authors used a read-based scRNA-seq technology. Correspondingly, the examination of the mean and empirical dropout rate revealed that the data followed a ZINB distribution (Supplementary Fig. 1C). Therefore, we denoised the 1000 most highly variable genes using DCA with ZINB noise model. Next, we performed differential expression analysis comparing H1 to DEC of the bulk and scRNA-seq data independently using DESeq2, which models gene expression based on the NB distribution without zero inflation^40^. After DCA denoising, 4 outlier genes (Fig. 5a, red dots), showing a high discrepancy between bulk and single-cell derived log fold changes, are corrected in the denoised data. *LEFTY1* is a key gene in the development of the endoderm^41,42^ and shows high expression in DEC compared to H1 in the bulk data (Fig. 5c). After DCA denoising, the median expression level of *LEFTY1* in DEC is shifted higher, more closely reflecting the observation in the bulk data (Fig. 5d, e).

Next, we systematically compared the four denoising methods for robustness using a bootstrapping approach. 20 random cells were sampled from H1 and DEC populations one hundred times and differential expression analysis using DESeq2 performed. When comparing the estimated log fold changes across all bootstrap iterations, DCA showed the highest correspondence with bulk log fold changes (Fig. 5f), indicating increased agreement between the DCA denoised and purified bulk data manifolds.

### Denoising increases protein and RNA co-expression

**Fig. 6:**
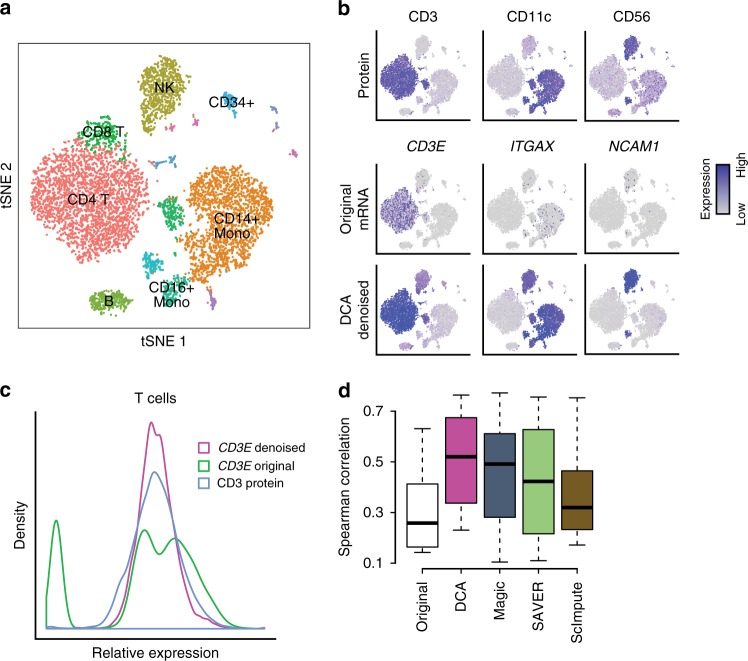
DCA increases protein and RNA co-expression. **a** depicts tSNE visualization of transcriptomic profiles of cord blood mononuclear cells from Stoeckius et al.^43^. Cells are colored by major immunological celltypes. **b** contains tSNE visualizations colored by protein expression (first row), RNA expression derived from the original (second row) and DCA denoised data (third row). Columns correspond to CD3 (first column), CD11c (second column), CD56 (third column) proteins and corresponding RNAs CD3E, ITGAX and NCAM1. **c** shows the distribution of expression values for CD3 protein (blue), original (green) and DCA denoised (pink) CD3E RNA in T cells. Spearman correlation coefficients for the eight protein-RNA pairs across all cells for the original and denoised data are plotted in **d**

CITE-seq enables simultaneous measurement of protein and RNA levels at cellular resolution. Per-cell protein levels are higher than mRNA levels for the corresponding genes and therefore less prone to dropout events^43^. Therefore, by using cell surface marker protein expressions as ‘ground truth’, denoising of mRNA levels can be evaluated. Stoeckius et al.^43^ used this CITE-seq method to profile cord blood mononuclear cells and identified major immunological celltypes (Fig. 6a). The original RNA count data was denoised using all four methods and evaluated. For DCA denoising the NB noise model was selected as the fits for NB and ZINB showed comparable goodness-of-fit (Supplementary Fig. 1E). Figure 6b shows tSNE visualization of the data colored by the expression levels of proteins CD3, CD11c, CD56 and corresponding RNAs *CD3E*, *ITGAX*, *NCAM1* by column, respectively. The rows correspond to the protein expression levels, RNA expression levels derived from the original and DCA denoised data. Visualizations for additional protein-mRNA pairs and other methods can be found in Supplementary Fig. 5 and 6, respectively. For example, the CD3 protein is expressed in 99.9% of T cells. The corresponding RNA *CD3E*, however, is only detected in 80% of T cells in the original count data. After denoising using DCA, *CD3E* is expressed in 99.9% of all T cells (Fig. 6c). Some slight discrepancies between the protein and denoised expression can be observed. For example, in the denoised data *ITGAX* shows expression in the natural killer cells (NK) cell cluster while the corresponding CD11c protein levels are very low. Checking data from the website of the Immunological Genome project (immgen.com) confirmed expression of *ITGAX* in NK cells, indicating that the denoised data for this gene reflects better agreement with known biology which may be obscured in the CITE-seq protein data due to some unknown technical reasons. To statistically evaluate the denoising methods we performed co-expression analysis using Spearman correlation for all eight available protein-mRNA pairs across all cells. DCA showed the highest median correlation coefficient, indicating that denoising increases protein and RNA co-expression (Fig. 6d).

### DCA runtime scales linearly with the number of cells

**Fig. 7:**
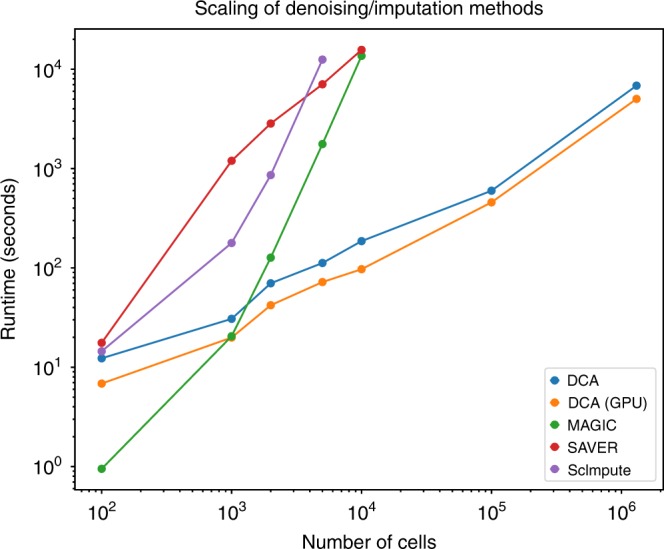
DCA scales linearly with the number of cells. Plot shows the runtimes for denoising of various matrices with different numbers of cells down-sampled from 1.3 million mouse brain cells^44^. Colors indicate different methods. DCA (GPU) indicates the DCA method run on the GPU

As the number of cells profiled in a single experiment is increasing, it is essential that scRNA-seq methods show good scalability. To assess the scalability of the four methods, we analyzed the currently largest scRNA-seq data set, consisting of 1.3 million mouse brain cells, from 10X Genomics. The 1.3 million cell data matrix was downsampled to 100, 1,000, 2,000, 5,000, 10,000 and 100,000 cells and 1000 highly variable genes. Each subsampled matrix was denoised and the runtime measured (Fig. 7). The runtime of DCA scaled linearly with the number of cells (slope = 0.66 for a linear fit on DCA points in log-log scale). While it took DCA minutes to denoise 100,000 cells, the other methods took hours. Therefore, DCA possesses a considerable speed advantage over the competing methods.

### Denoising enables discovery of subtle cellular phenotypes

**Fig. 8:**
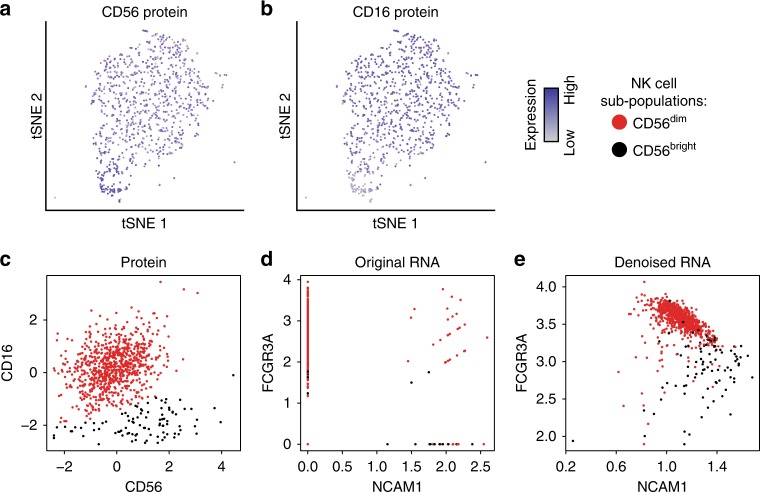
Denoising enhances discovery of cellular phenotypes. tSNE visualization of transcriptomically derived NK cell cluster colored by CD56 (**a**) and CD16 (**b**) protein expression levels. Grey and blue indicate relative low and high expression, respectively. **c** shows CD56 and CD16 protein expression across NK cells, revealing two distinct sub-populations defined as CD56dim (red) and CD56bright (bright). **d**, **e** depict expression of corresponding RNAs NCAM1 and FCGR3A using the original count data and DCA denoised data, respectively. Cells are colored by protein expression derived assignment to CD56bright (black) and CD56dim (red) NK cell sub-populations

After having evaluated DCA against competing methods, we tested if DCA denoising could enhance biological discovery which is impossible or more challenging to obtain without denoising. Stoeckius et al^43^. highlight the potential for integrated and multimodal analyses to enhance the discovery of cellular phenotypes, particularly when differentiating between cell populations with subtle transcriptomic differences. The authors observed an opposing gradient of CD56 and CD16 protein levels within the transcriptomically derived NK cell cluster (Fig. 8a, b). Indeed, unsupervised clustering using Gaussian mixture model on the CD16 and CD56 protein expression levels revealed two sub-populations of cells (Fig. 8c). The corresponding RNAs *NCAM1* and *FCGR3A*, however, contained high levels of dropout obscuring the protein derived sub-population structure (Fig. 8d). After denoising, the two sub-populations of NK cells become visually more clearly evident based on DCA denoised *NCAM1* and *FCGR3A* RNA expression levels (Fig. 8e). To assess the agreement between the protein-derived sub-population structure and the expression data, we calculated the silhouette coefficients based on the Euclidean distance of the expression derived from the protein, original and denoised data (Average Silhouette widths: 0.47, 0.17, 0.58, respectively), which demonstrated higher correspondence between the protein and denoised compared to the original RNA data. Therefore, DCA denoising enabled the extraction of information which was exclusively contained in the CITE-seq proteins, demonstrating the ability to enable the discovery of subtle cellular phenotypes.

### Denoising increases correlation structure of key regulators

**Fig. 9:**
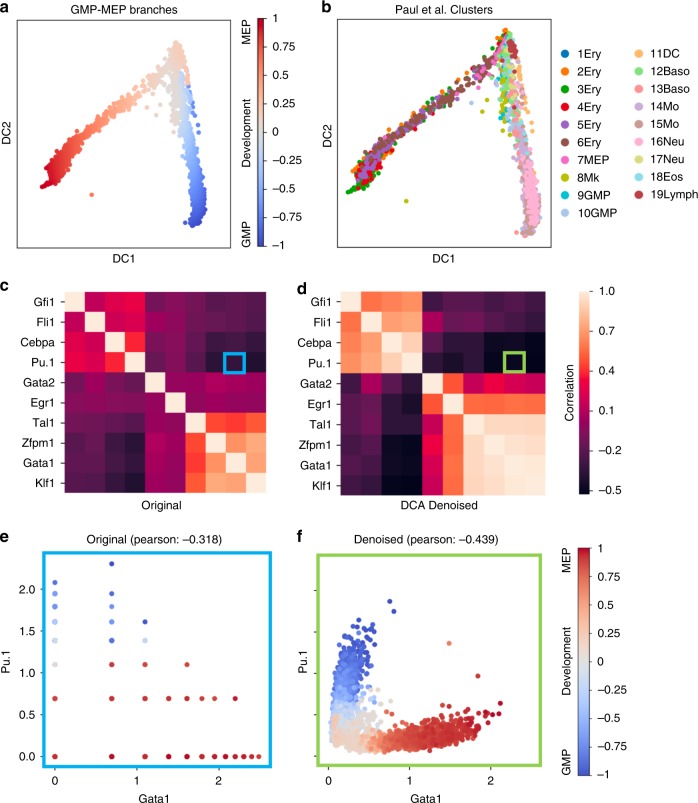
Denoising by DCA increases correlation structure of key regulatory genes. **a**, **b** display diffusion maps of blood development into GMP and MEP colored by developmental trajectory and celltype, respectively. Abbreviations Ery, Mk, DC, Baso, Mo, Neu, Eos, Lymph correspond to erythrocytes, megakaryocytes, dendritic cells, basophils, monocytes, neutrophils, eosinophils and lymphoid cells, respectively. **c**, **d** display heatmaps of correlation coefficients for well-known blood regulators taken from Krumsiek et al.^45^. Highlighted areas show *Pu.1* - *Gata1* correlation in the heatmap. **e**, **f** show anti-correlated gene expression patterns of *Gata1* and *Pu.1* transcription factors colored by pseudotime, respectively

Next, we tested if denoising enhances discovery of regulatory relationships for well-known transcription factors in blood development^44^. As previously mentioned, in Paul et al.^35^ the authors describe the transcriptional differentiation landscape of blood development into MEP and GMP (Fig. 9a, b). After denoising, a set of well-known MEP and GMP regulators^45^ show enhanced regulatory correlations (Fig. 9c, d), for example, the anticorrelation between *Pu.1* and *Gata1* increases (Fig. 9e, f). These two transcription factors are important regulators in blood development and known to inhibit each other^46^. This regulatory relationship is identified in denoised data also in cells with zero expression for either gene in the original data, demonstrating the ability of DCA to extract meaningful information from otherwise non-informative zero count values (Supplementary Fig. 5). Overall, these results demonstrate that DCA enhances the modeling of gene regulatory correlations, and we expect future network inference methods to use denoising as a first preprocessing step.

### Evaluation of hyperparameter selection

**Fig. 10:**
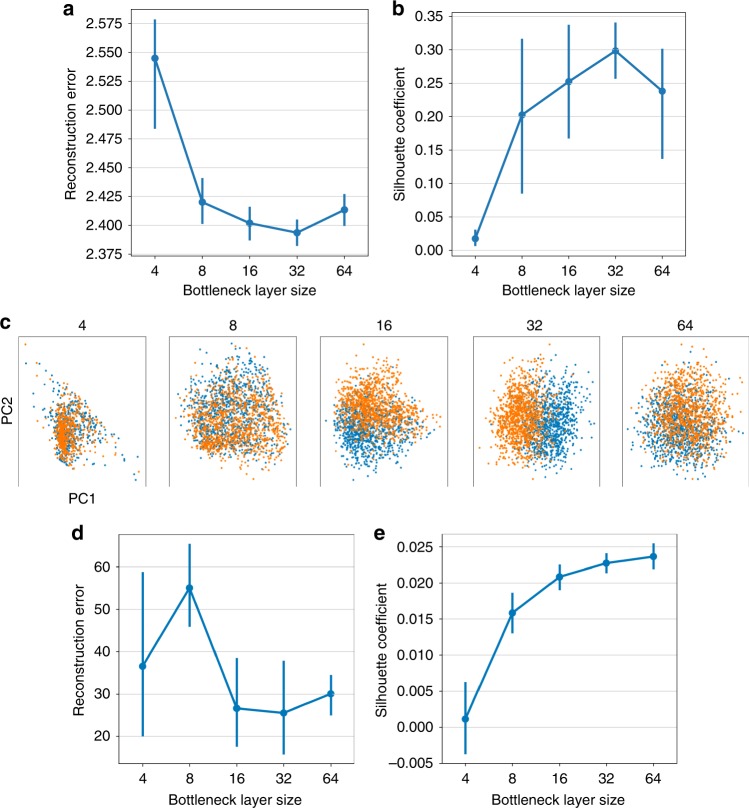
ReconstructionTraining error correlates with DCA performance and can guide hyperparameter selection. **a**, **b** show the distribution of the reconstructiontraining error and Silhouette coefficients across five different bottleneck layer sizes, respectively. Error bars represent standard error across five iterations. **c** shows exemplary PCA results derived from denoised expression data across the five bottleneck layer configurations. Colors represent simulated celltypes. **d**, **e** show the distribution of the reconstruction error and Silhouette coefficients when applying analogous analysis to the Zheng et al.^12^ data

The choice of the noise model represents the only parameter the user has to specify. As previously mentioned, we describe an approach to guide the user in the selection of the noise model. Additionally, our DCA framework provides a large set of hyperparameters for tuning the model. To assess the impact of hyperparameter choice on the performance of DCA and to provide guidance to users we conducted the following analyses. We denoised the two group simulation data varying the size of the bottleneck layer. We tested five different bottleneck layer sizes (4, 8, 16, 32 and 64 neurons) and performed DCA denoising five times per size. During each iteration the final reconstruction error was saved, PCA performed on the denoised output and the Silhouette coefficient assessing the celltype clustering structure was calculated. Low reconstruction error indicates a good hyperparameter configuration, while high Silhouette coefficient indicates a good separation between the celltypes. The reconstruction error (Fig. 10a) and silhouette coefficient (Fig. 10b) show the minimum and maximum values at a bottleneck layer size of 32 neurons, respectively. Selecting too low or high dimensional bottleneck layer sizes decreases the performance of DCA as measured in the ability to separate the two simulated celltypes (Fig. 10c). Analogous results were obtained when applying this analysis scheme to real data. We denoised the Zheng et al.^12^ data varying the bottleneck layer configuration as described above and calculated the Silhouette coefficient based on the Euclidean distance of the principal components and the original celltype labels from Zheng et al.^12^ (Fig. 10d, e). The agreement between the DCA intrinsic reconstruction error and the downstream evaluation in both simulated and read data indicates that the reconstruction error can be used to guide hyperparameter selection. Therefore, DCA implements an automated hyperparameter search which identifies the set of hyperparameters that minimizes the reconstruction error.

## Discussion

One of the fundamental challenges in scRNA-seq analysis is technical variation. Recent research has shown that accounting for technical variation improves downstream analysis^7,47–49^ such as uncovering the cell differentiation structure, identification of highly variable genes, and clustering. Furthermore, some denoising/imputation methods have been implemented in scRNA-seq workbenches such as Granatum^50^, indicating that it is an important, frequently used processing or smoothing step e.g. for visualization.

Here, we introduce a robust and fast autoencoder-based denoising method tailored to scRNA-seq datasets, which represents one of the first applications of deep learning to scRNA-seq data. We demonstrate that denoising scRNA-seq data can remove technical variation improving five possible downstream analyses, namely clustering, time course modeling, differential expression, protein-RNA co-expression and pseudotime analyses. Furthermore, we show that DCA is highly scalable to datasets with up to millions of cells.

The evaluation of denoising is difficult because the definition of a ground truth can be challenging for real data. We, therefore, described a diverse set of evaluation scenarios, which may allow systematic assessment of other denoising techniques in the future. Furthermore, in order to avoid bias in comparisons, we adapted evaluation approaches and used corresponding data from competing methods for evaluation.

Note that in general, it may be difficult to determine when denoising improves scRNA-seq data. As expected, we observe increased gene-gene correlation after denoising; while in our examples this enriched for desired regulatory dependencies, this may also lead to overimputation in case of inadequate hyperparameter choices such as too low-dimensional bottleneck layer and hence data manifold. To alleviate overfitting and overimputation, a general and not yet extensively treated issue of imputation methods, we implemented a number of regularization methods, including dropout, encoder-specific and overall L1 and L2 regularization. This is required especially when training on data sets with limited sample size. DCA also allows users to conduct a hyperparameter search to find the optimal set of parameters for denoising to avoid poor generalization due to overfitting. However, we would like to point out that hyperparameters were not fine-tuned for any of the analyses described in the manuscript. Additionally, DCA enables parallelization using GPUs.

The proposed method can be easily integrated into existing workflows; in particular, it supports h5ad-formatted HDF5 files (https://github.com/theislab/anndata) and the Python API is compatible with the Scanpy^33^ package.

## Methods

### Autoencoders

Artificial neural networks were shown to outperform traditional approaches as they learn complex structure in the data to predict an outcome^51,^. A specialization is an “autoencoder” when no outcome information is available. An autoencoder learns to predict input data using three layers: an input layer, a hidden (“bottleneck”) layer and an output layer^23^. It is characterized by the fact that both input and output layers are of the same size (i.e. same number of genes) and the bottleneck layer is of much lower dimensionality. By adjusting the weights of the neural network, the autoencoder learns in an unsupervised manner how to efficiently compress and subsequently reconstruct the data using typically MSE loss function. Since the compression forces the autoencoder to learn only the essential latent features, the reconstruction ignores non-essential sources of variation such as random noise (Fig. 1a). Therefore, the compressed representation reflects the high dimensional ambient data space in significantly lower dimensionality and captures the underlying true data manifold. For example, in a data set where snapshots of differentiating blood cells exist, the manifold captures the continuum of differentiation phenotypes^17^ in a zero-noise scenario. For an analogy, principal component analysis (PCA) can be interpreted as a linear autoencoder with MSE loss function. Reconstruction of the data with the first two principal components corresponds to the output of a linear autoencoder with a two-dimensional bottleneck layer.

### Noise model

The ZINB distribution models highly sparse and overdispersed count data. The ZINB mixture model consists of the following two components: (1) a point mass at zero which represents excess zero values in the data and (2) a negative binomial component representing the count distribution. For scRNA-seq data, the point mass at zero may capture dropout events while the negative binomial component of the distribution represents the process of sampling reads from underlying molecules.

The ZINB distribution is parameterized with mean and dispersion parameters of the negative binomial component (*μ* and *θ*) and the mixture coefficient that represents the weight of the point mass (*π*):

1 $${\it{{\rm{NB}}}}\left( {{\it{x}};{\it{\mu }},{\it{\theta }}} \right) = \frac{{{\it{\Gamma }}\left( {{\it{x}} + {\it{\theta }}} \right)}}{{{\it{\Gamma }}\left( {\it{\theta }} \right)}}\left( {\frac{{\it{\theta }}}{{{\it{\theta }} + {\it{\mu }}}}} \right)^{\it{\theta }}\left( {\frac{{\it{\mu }}}{{{\it{\theta }} + {\it{\mu }}}}} \right)^{\it{x}}$$

2 $${{\text{ZINB}}}\left( {{\it{x}};{\it{\pi }},{\it{\mu }},{\it{\theta }}} \right) = {\it{\pi \delta }}_0\left( {\it{x}} \right) + \left( {1 - {\it{\pi }}} \right){\text{NB}}\left( {{\it{x}};{\it{\mu }},{\it{\theta }}} \right)$$

### Architecture and training

Here we use the autoencoder framework to estimate three parameters of ZINB distribution conditioned on the input data for each gene. Therefore, unlike traditional autoencoders, our model also estimates dropout (*π*) and dispersion (*θ*) parameters in addition to the mean (*μ*). Each module corresponds to a parameter of the ZINB distribution, given as *μ*, *θ* and *π*. In this setting, the size of the input layer and three output layers corresponding to these parameters have the same number of features (genes). However, unlike typical autoencoders, there are three output layers instead of one, representing for each gene the three parameters (*μ*, *θ*, *π*) that make up the gene-specific loss function to compare to the original input of this gene. For an analogy, in binary classifiers, the output layer is interpreted as logistic regression using the features extracted from the previous layers. Similarly, the output layer in our approach can be interpreted as ZINB regression where predictors are new representations of cells.

The formulation of the architecture is given below:

3 $$\begin{array}{rcl}{\boldsymbol{E}} &= {\it{{\rm{ReLU}}}}\left( {{\bar{\boldsymbol X}}{\boldsymbol{W}}_{\it{E}}} \right)\\ {\boldsymbol{B}} &= {\it{\rm{ReLU}}}\left( {{\boldsymbol{EW}}_{\it{B}}} \right)\\ {\boldsymbol{D}} &= {\it{{\rm{ReLU}}}}\left( {{\boldsymbol{BW}}_{\it{D}}} \right)\\ {\bar{\boldsymbol M}} &= {\it{{\rm{exp}}}}\left( {{\boldsymbol{DW}}_{\it{\mu }}} \right)\\ {\boldsymbol{{\Pi}}} &= {\it{{\rm{sigmoid}}}}\left( {{\boldsymbol{DW}}_{\it{\pi }}} \right)\\ {\boldsymbol{\Theta }} &= {\it{{\rm{exp}}}}\left( {{\boldsymbol{DW}}_{\it{\theta }}} \right)\end{array},$$

where **E**, **B** and **D** represent the encoder, bottleneck and decoder layers, respectively. In this formulation, $\mathop {{\mathbf{X}}}\limits^ -$ represents library size, log and *z* score normalized expression matrix, where rows and columns correspond to cells and genes, respectively. Size factors for every cell, *s*_*i*_, is calculated as the total number of counts per cell divided by the median of total counts per cell. $\mathop {{\mathbf{X}}}\limits^ -$ is defined as:

4 $${\bar{\boldsymbol X}} = {\it{{\rm{zscore}}}}({\it{{\rm{log}}}}({\rm{diag}}(s_i)^{ - 1}{\boldsymbol{X}} + 1))$$

where **X** and "zscore" represent the raw count matrix and z-score normalization.

Output activations are shown here in matrix form as ${\bar{\mathbf M}}$, *Θ* and ∏. Although the mini-batch stochastic gradient descent is used for optimization, for clarity we depict the matrices of size *n*×*p* where n and p represent the number of cells and genes, respectively.

The activation function of the mean and dispersion output layers is exponential since the mean and dispersion parameters are always non-negative. The third output ∏ estimates the dropout probability for every element of the input. The activation function of this layer is sigmoid as ∏ values represent the dropout probabilities and are therefore limited to the range between zero and one. The activation function of the three output layers is an inverse canonical link function of a ZINB regression model in the context of generalized linear models.

The loss function represents the likelihood of the ZINB distribution:

5 $$\begin{array}{rcl}{\hat{\boldsymbol {\Pi}}},{\hat{\boldsymbol M}},{\hat{\boldsymbol \Theta }} &= {\it{{\rm{argmax}}}}_{{\boldsymbol{{\Pi}}},{\boldsymbol{M}},{\boldsymbol{\Theta }}}{\it{{\rm{ZINB}}}}\left( {{\boldsymbol{X}};{\boldsymbol{{\Pi}}},{\boldsymbol{M}},{\boldsymbol{\Theta }}} \right)\\ &= {\it{{\rm{argmax}}}}_{{\boldsymbol{{\Pi}}},{\boldsymbol{M}},{\boldsymbol{\Theta }}}\mathop {\prod }\limits_{{\boldsymbol{i}} = 1}^{\boldsymbol{n}} \mathop {\prod }\limits_{{\boldsymbol{j}} = 1}^{\boldsymbol{p}} {\it{{\rm{ZINB}}}}({\it{x}}_{{\it{ij}}};{\it{\pi }}_{{\it{ij}}},{\it{\mu }}_{{\it{ij}}},{\it{\theta }}_{{\it{ij}}})\end{array},$$

where *x*_*ij*_ represents the elements in the raw count matrix **X**, *i* and *j* represent cell and gene indices and n and p represent the number of cells and genes. **M** represents the mean matrix multiplied by the size factors that are calculated before the training:

6 $${\boldsymbol{M}} = {\it{{\rm{diag}}}}\left( {{\it{s}}_{\it{i}}} \right)\mathop {{\boldsymbol{M}}}\limits^ -,$$

which keeps the hidden representation of cells and the optimization process independent of library size differences.

Furthermore, our model contains a tunable zero-inflation regularization parameter that acts as a prior on the weight of the dropout process. This is achieved using the ridge prior on the dropout probabilities and zero inflation (∏ parameter):

7 $$\begin{array}{rcl}{\hat{\boldsymbol {\Pi}}},{\hat{\boldsymbol M}},{\hat{\boldsymbol \Theta }} &= {\mathrm{argmin}}_{{\boldsymbol{{\Pi}}},{\boldsymbol{M}},{\boldsymbol{\Theta }}}{\text{NLL}}_{{\text{ZINB}}}\left( {{\boldsymbol{X}};{\boldsymbol{{\Pi}}},{\boldsymbol{M}},{\boldsymbol{\Theta }}} \right) + {\it{\lambda }}\left\| {\boldsymbol{{\Pi}}} \right\|_{\it{F}}^2\\ & = {\mathrm{argmin}}_{{\boldsymbol{{\Pi}}},{\boldsymbol{M}},{\boldsymbol{\Theta }}}\mathop {\sum }\limits_{{\boldsymbol{i}} = 1}^{\boldsymbol{n}} \mathop {\sum }\limits_{{\boldsymbol{j}} = 1}^{\boldsymbol{p}} {\text{NLL}}_{{\text{ZINB}}}({\it{x}}_{{\it{ij}}};{\it{\pi }}_{{\it{ij}}},{\it{\mu }}_{{\it{ij}}},{\it{\theta }}_{{\it{ij}}}) + {\it{\lambda \pi }}_{{\it{ij}}}^2\end{array},$$

where NLL_ZINB_ function represents the negative log likelihood of ZINB distribution.

To increase flexibility, we provide implementations of NB, ZINB, Poisson and MSE noise models. Furthermore, users are also allowed to choose whether the dispersion parameter is conditioned on the input. While n *x* *p* dispersion matrix is estimated from the data in the conditional dispersion (default option), the alternative option estimates an independent dispersion parameter per gene.

### Hyperparameter search

Hyperparameter search allows users to find optimal *λ* value for a given data set along with other hyperparameters like hidden layer configuration, type of activation function, and the strength of L1/L2 regularization on the parameters. For the hyperparameter search, DCA is trained with one thousand hyperparameter configurations sampled from specified ranges for each hyperparameter and the hyperparameter configuration with the lowest reconstruction error is selected. Tree-structured Parzen Estimator (TPE) method implemented in hyperopt^52^ is used as the optimization method.

### Zero inflation analysis

To select a suitable noise model, we fit NB and ZINB models to the mean and empirical dropout rate dependence by minimizing the binary cross entropy (BCE) between the observed and predicted dropout rates. For the NB fit, the dispersion parameter is optimized, while for the ZINB model, the zero-inflation parameter (*π*) is modelled as an affine transformation of the observed mean. Therefore, in addition to the dispersion, two more parameters, the slope and the offset are jointly optimized to minimize the BCE. Finally, log-likelihood ratio test is performed using the difference between the negative BCE values of model fits.

### Denoising

The denoised matrix is generated by replacing the original count values with the mean of the negative binomial component (${\bar{\mathbf M}}$ matrix in Equation 3) as predicted in the output layer. This matrix represents the denoised and library size normalized expression matrix, the final output of the method. Intuitively, our approach can be interpreted as a two-step process. First, the data is summarized by extracting lower dimensional hidden features that are useful for denoising the data as well as identifying and correcting dropout zeros. Then, a ZINB regression is fitted using these new hidden features. However, these two steps are performed simultaneously during the training.

### Implementation

DCA is implemented in Python 3 using Keras^53^ and its TensorFlow^54^ backend. We used RMSProp for optimization with learning rate 0.001. Learning rate is multiplied by 0.1 if validation loss does not improve for 20 epochs. The training stops after no improvement in validation loss for 25 epochs. Gradient values are clipped to 5 and the batch size is set to 32 for all datasets. All hidden layers except for the bottleneck consist of 64 neurons. The bottleneck has 32 neurons. Training on CPU or GPU is supported using Keras and TensorFlow.

The hyperparameter search is implemented using hyperopt and kopt Python packages (https://github.com/Avsecz/kopt).

### Simulated scRNA-seq data

Simulated datasets were generated using the Splatter R package^34^. For the two group simulation the following parameters were used in the *splatSimulate()* R function: groupCells = 2, nGenes = 200, dropout.present = TRUE, dropout.shape -1, dropout.mid = 5. For the six group simulation the following parameters were used in the *splatSimulate()* R function: groupCells = 6, nGenes = 200, dropout.present = TRUE, dropout.shape -1, dropout.mid = 1.

### 68k peripheral blood mononuclear cell experiment

Single-cell gene expression count matrix and celltype labels from Zheng et al. were downloaded from http://www.github.com/10XGenomics/single-cell-3prime-paper. Since CD4+ and CD8+ subtype clusters are highly overlapping, they are combined into coarse groups. tSNE coordinates were obtained by reproducing the code from single-cell-3prime-paper repository. For the population structure analysis (Fig. 3), DCA was run using the following parameter: -s 16,2,16. For the hyperparameter search, various bottleneck layer sizes are compared using -s 64,*i*,64, where *i* represents the bottleneck size being tested.

### MAGIC

MAGIC (version 0.1.0) was downloaded from https://github.com/pkathail/magic. MAGIC was run using default parameters specified as 20 for the numbers of principal components, 6 for the parameter t for the power of the Markov affinity matrix, 30 the number of nearest neighbors, 10 the autotune parameter and 99th percentile to use for scaling.

### scImpute

scImpute (version 0.0.5) was downloaded from https://github.com/Vivianstats/scImpute. For the C. elegans development experiment, the Chu et al^39^. definitive endoderm differentiation experiment, the CITE-seq cord blood mononuclear cells experiment and the scalability analysis, kCluster = 1, kCluster =2, kCluster = 13 and kCluster = 2 parameters were used, respectively.

### SAVER

SAVER (version 0.3.0) was downloaded from https://github.com/mohuangx/SAVER. SAVER was run using default parameters specified as 300 for the maximum number of genes used in the prediction, 50 for the number of lambda to calculate in cross-validation and 5 for the number of folds used in cross-validation. For the scalability analysis, SAVER was run using the R package *doParallel* with 24 cores.

### DCA

For the two and six group simulation data, *C*. *elegans* development and the Chu et al.^39^ definitive endoderm differentiation experiments the DCA default parameters were used. For the zero-count analysis, DCA was run using the --ridge 0.005 hyperparameter. This hyperparameter penalizes model complexity by shrinking inferred dropout probabilities (*π*). For the CITE-seq cord blood mononuclear cells experiment, Paul et al. early blood development experiment and the 68k peripheral blood mononuclear cell experiment following parameters were used -*-type nb*.

### C. elegans development experiment

Francesconi et al^36^. data set contained 206 samples covering a 12-hour time-course. Similar to the evaluation proposed van Dijk et al^20^., expression values were exponentiated to create a count distribution and subsequently single-cell noise was added in silico by subtracting gene-specific artificial noise from each gene. Gene-specific artificial noise was generated using the exponential function where the mean was calculated as the gene expression median multiplied by five. Any negative values were set to zero so that on average 80% of the values were zero. Pearson correlation was calculated between the expression level of each gene and time course to identify top 500 development genes.

### Definitive endoderm differentiation experiment

The gene expression data from Chu et al.^39^ was restricted to single cells and bulk samples from H1 and DEC using the provided annotation and the 1000 most highly variable genes. Differential expression analysis was performed using the R package DESeq2 (version 1.14.1). DESeq2 models gene expression based on a negative binomial distribution without zero-inflation. To retain count structure, denoised data for all methods was rounded prior to analysis. The dispersion was estimated using “mean” for the *fitType* parameter. To assess the robustness of the results, bootstrapping analysis was conducted. During each of 100 iterations, 20 cells from the H1 and DEC cells were randomly selected and differential expression analysis performed as described above. Next, concordance was evaluated using the Pearson correlation between the estimated fold changes derived from the single-cell bootstrap and bulk data.

### CITE-seq cord blood mononuclear cells experiment

The Seurat R package was used to perform the analysis. Following the instructions of the authors^43^ data were subset to 8,005 human cells by removing cells with less than 90% human UMI counts. Next, RNA data were normalized, highly variables genes were identified and expression data was scaled. First 13 principal components were calculated and used for clustering and tSNE visualization. A total of 13 clusters were identified. The *genesCLR* method was used for normalization of the protein data. For denoising, gene expression data was restricted to the top 5000 highly variable genes. Co-expression for eight known marker proteins (CD3, CD19, CD4, CD8, CD56, CD16, CD11c, CD14) and corresponding mRNAs (*CD3E, CD19, CD4, CD8A, NCAM1, FCGR3A, ITGAX, CD14*) was assessed using Spearman correlation on the scaled expression data across all 8,005 cells. It is important to note, that the correlation is calculated across all cells and not within a single celltype. Therefore, the correlation coefficient will capture the presence and absence of protein and mRNA more so than a direct linear dependency between the expression levels of the two.

### NK subset analysis

Stoeckius et al^43^. data were subset to 906 NK cells. Next, protein and RNA expression data were scaled. Using CD16 and CD56 protein expression levels, cells were clustered with the *Mclust()* function from the R *mclust* package and two mixture components. To quantitatively assess the correspondence between protein derived sub-populations and mRNA expression levels, the Silhouette coefficient was calculated. The Silhouette coefficient ranges from -1 to 1 and values close to zero indicate random clustering with regards to the specified indicator.

### Blood regulator analysis

Paul et al. blood differentiation data with 2730 cells and 3451 informative genes are used for the analysis. After log transformation with a pseudo-count of one, the kNN graph is constructed using the “*scanpy.api.pp.neighbors*” function. Diffusion map, diffusion pseudotime (DPT) and four diffusion pseudotime groups are computed with “*scanpy.api.tl.dpt(adata, n_branchings=1)*”. Pseudotime estimates of the two DPT groups corresponding to MEP and GMP branches are scaled between [0, 1] and [0, -1] in order to show the branching more distinctly. DCA is run with default parameters and Pearson correlation coefficients between marker genes are calculated with “*numpy.corrcoef*” function. For the 2-neuron bottleneck analysis, DCA was run using the following parameter: -s 16,2,16.

### Scalability analysis

First, cells and genes with zero expression are removed from the count matrix. Next, the top 1000 highly variable genes are selected using “filter_genes_dispersion” function of Scanpy with n_top_genes=1000 argument. The 1.3 million cell data matrix was downsampled to 100, 1000, 2000, 5000, 10,000 and 100,000 cells and these 1000 highly variable genes. Each subsampled matrix was denoised using the four methods and the runtimes measured. Scalability analysis was performed on a server with two Intel Xeon E5-2620 2.40 GHz CPUs. NVIDIA GeForce GTX TITAN X is used for denoising datasets on GPU with DCA.

### Code availability

DCA, including usage tutorial and code to reproduce the main figures in the manuscript, can be downloaded from https://github.com/theislab/dca.

## Supplementary information

Supplementary Information

Reporting Summary

## Data Availability

The data sets analysed during the current study are publicly available. Bulk microarray gene expression of developing C.elegans embryos was downloaded the supplementary material of Francesconi et al^36^. Chu et al.^39^ single-cell and bulk RNA-seq data for definitive endoderm differentiation experiment are available at the Gene Expression Omnibus (GEO) under accession code GSE75748. Single-cell protein and RNA raw count expression matrices for CITE-seq cord blood mononuclear cells experiment are available at GEO under accession code GSE100866. Paul et al. blood differentiation data including the celltype annotations are obtained via “*scanpy.api.datasets.paul15()”* function of Scanpy Python package. 1.3 million mouse brain cell data were downloaded from https://support.10xgenomics.com/single-cell-gene-expression/datasets/1.3.0/1M_neurons. 68k PBMC data from Zheng et al.^12^ is downloaded from http://www.github.com/10XGenomics/single-cell-3prime-paper.
