# Supplementary material for: Single-cell RNA-seq denoising using a deep count autoencoder
Source: Nat Commun. 2019 Jan 23;10:390. doi: 10.1038/s41467-018-07931-2 (PMC6344535; doi:10.1038/s41467-018-07931-2)
Supplement: Supplementary file 1 — Supplementary Information [file 41467_2018_7931_MOESM1_ESM.pdf]

Supplementary Information

Single cell RNA-seq denoising using a deep count autoencoder

Gökcen Eraslan*, Lukas M. Simon*, Maria Mircea, Nikola S. Mueller, Fabian J. Theis

*authors contributed equally

Correspondence to fabian.theis@helmholtz-muenchen.de

Supplementary Figure 1

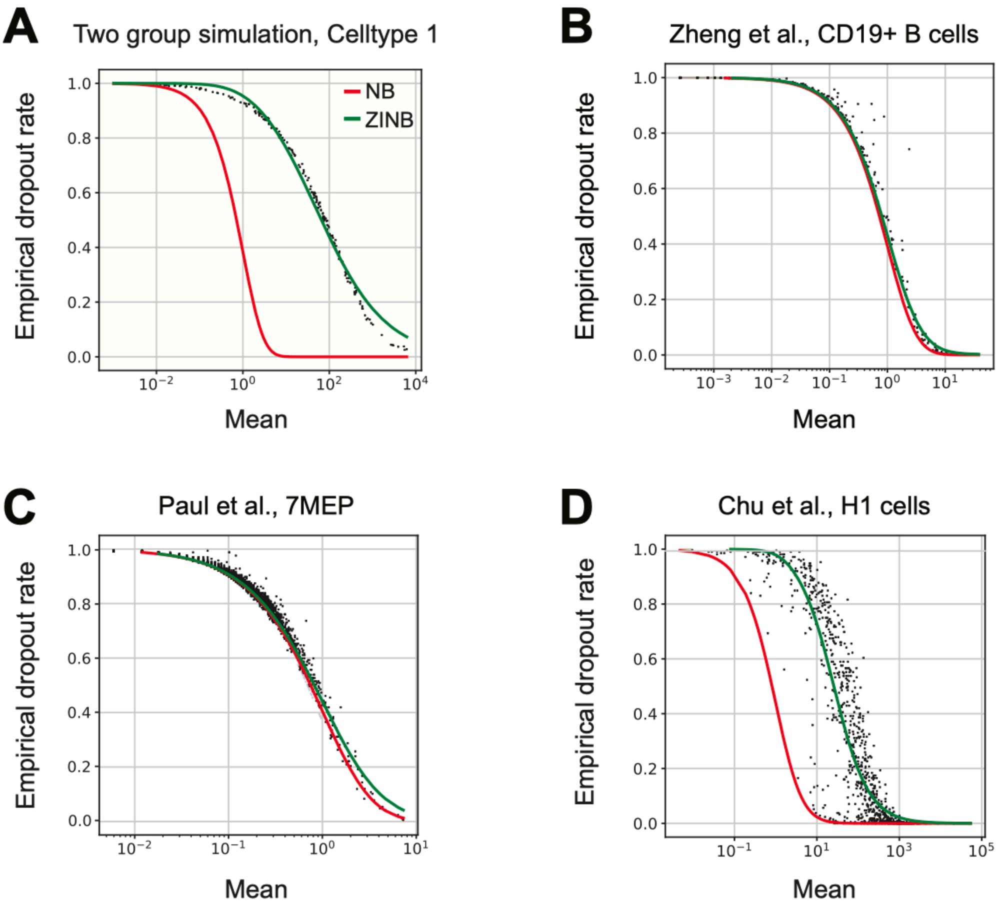

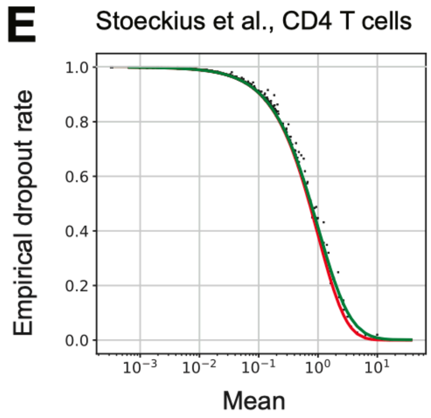

F

Dataset	Cluster	Type	BCE_{NB}	BCE_{ZINB}	p-value
**Two group simulation**	Celltype 1	NA	878.11	90.79	< 1e-16
Zheng et al.	CD19+ B cells	UMI	85.60	84.70	0.405
Paul et al.	7MEP	UMI	890.48	889.78	0.498
**Chu et al.**	H1 cells	read	1299.72	348.41	< 1e-16
Stoeckius et al.	CD4 T cells	UMI	269.64	269.47	0.844

Supplementary Figure 1. Panels A-E show the mean and empirical dropout rate on the X and Y axis for the data sets analyzed throughout the manuscript, respectively. Panel F summarizes the negative log likelihood estimates and p-values of the likelihood ratio tests between NB and ZINB for each of the data sets. In agreement with Chen et al., the likelihoods and p-values indicate that UMI based scRNA-seq technologies do not show zero inflation.

Supplementary Figure 2

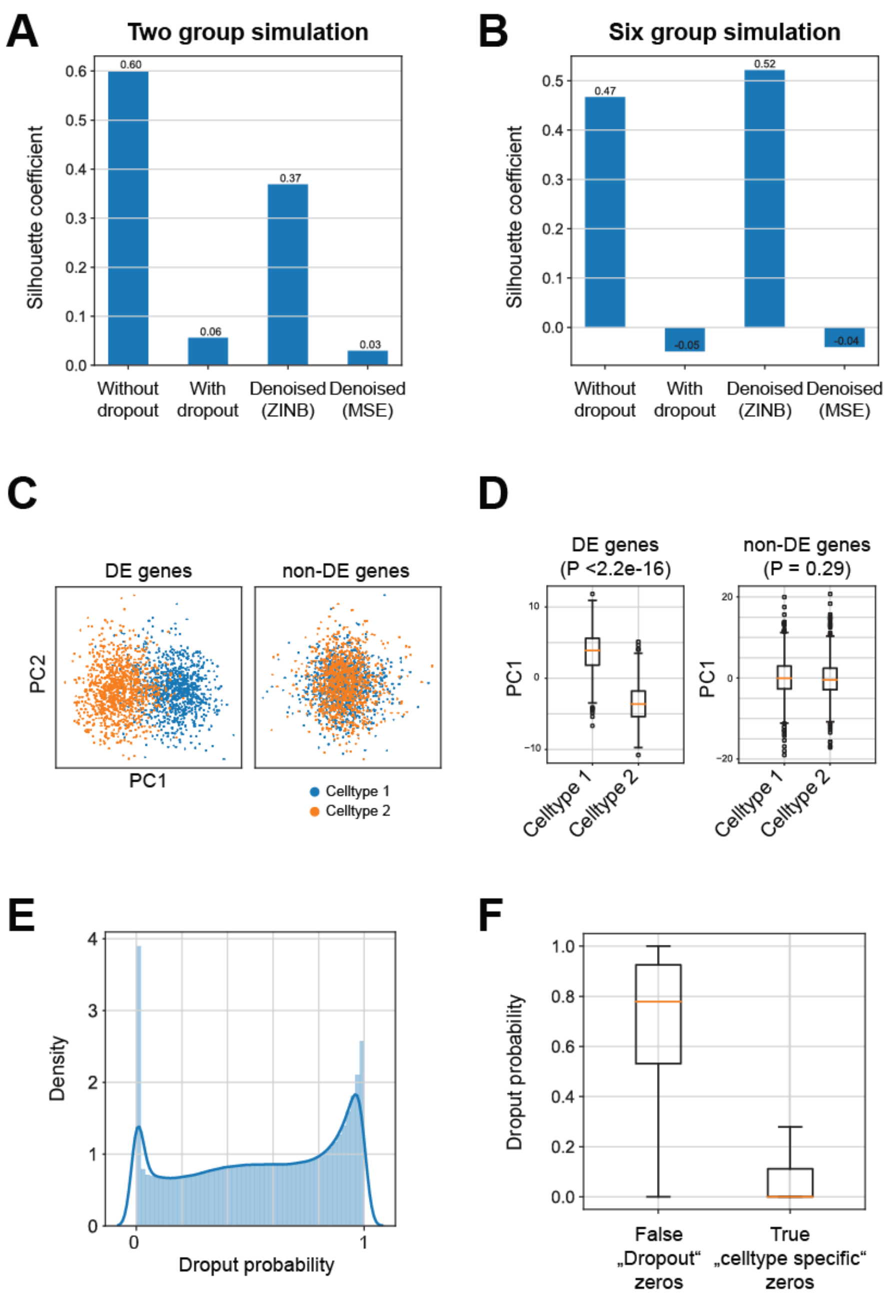

Supplementary Figure 2. Panels A and B depict the Silhouette coefficient calculated on the celltype assignment and PCA or tSNE space for the two and six group simulations, respectively. DCA denoising recovers celltype clustering in contrast to MSE based autoencoder. Panel C shows the PCA plot for two group simulation data when restricting feature space to DE (left) and non-DE (right) genes. PCA based on DE genes shows significant association between PC 1 and celltype assignment (Panel D, left). However, non-DE genes show no significant association (Panel D, right), indicating that celltype clustering is not recovered and DCA

denoising is robust to overfitting. Panel E illustrates the distribution of inferred dropout probabilities as captured by the dropout (π) parameter. Panel F shows the inferred dropout probabilities for false “dropout” and true “celltype specific” zero entries.

Supplementary Figure 3

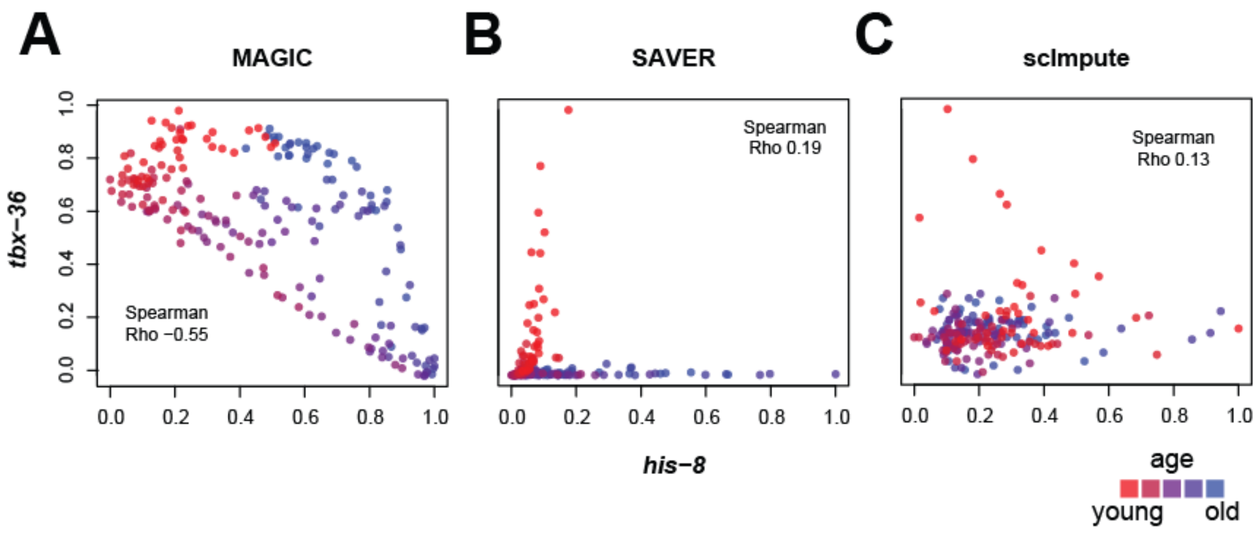

Supplementary Figure 3. Scatter plots illustrate gene expression levels for exemplary gene pair *tbx-36* and *his-8* over the developmental time course for data denoised with SAVER (Panel A), scImpute (Panel B) and MAGIC (Panel C).

Supplementary Figure 4

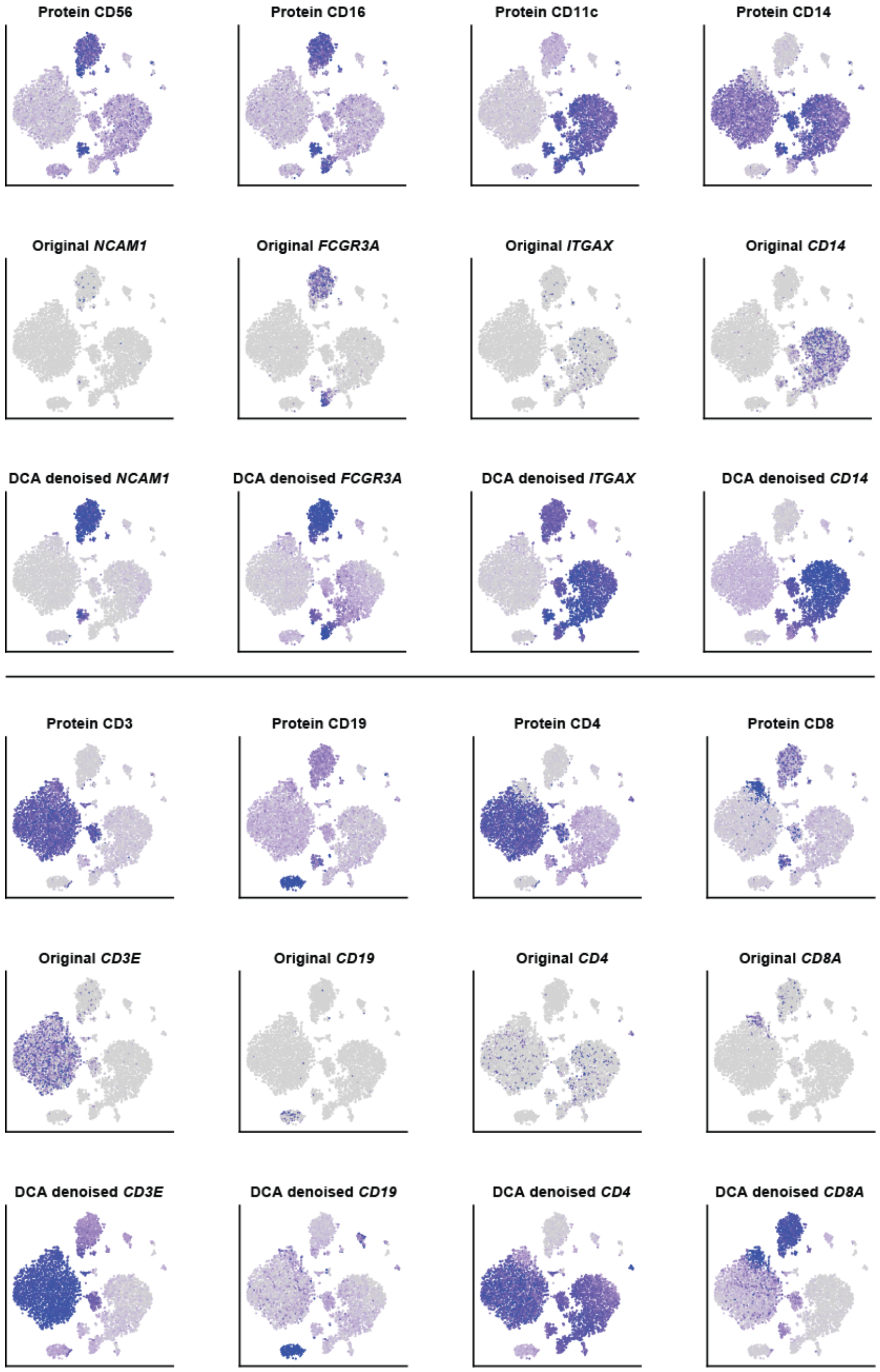

Supplemental Figure 4. tSNE visualizations colored by protein expression (first row), RNA expression derived from original (second row) and DCA denoised data (third row) for all eight protein-RNA pairs.

Supplementary Figure 5

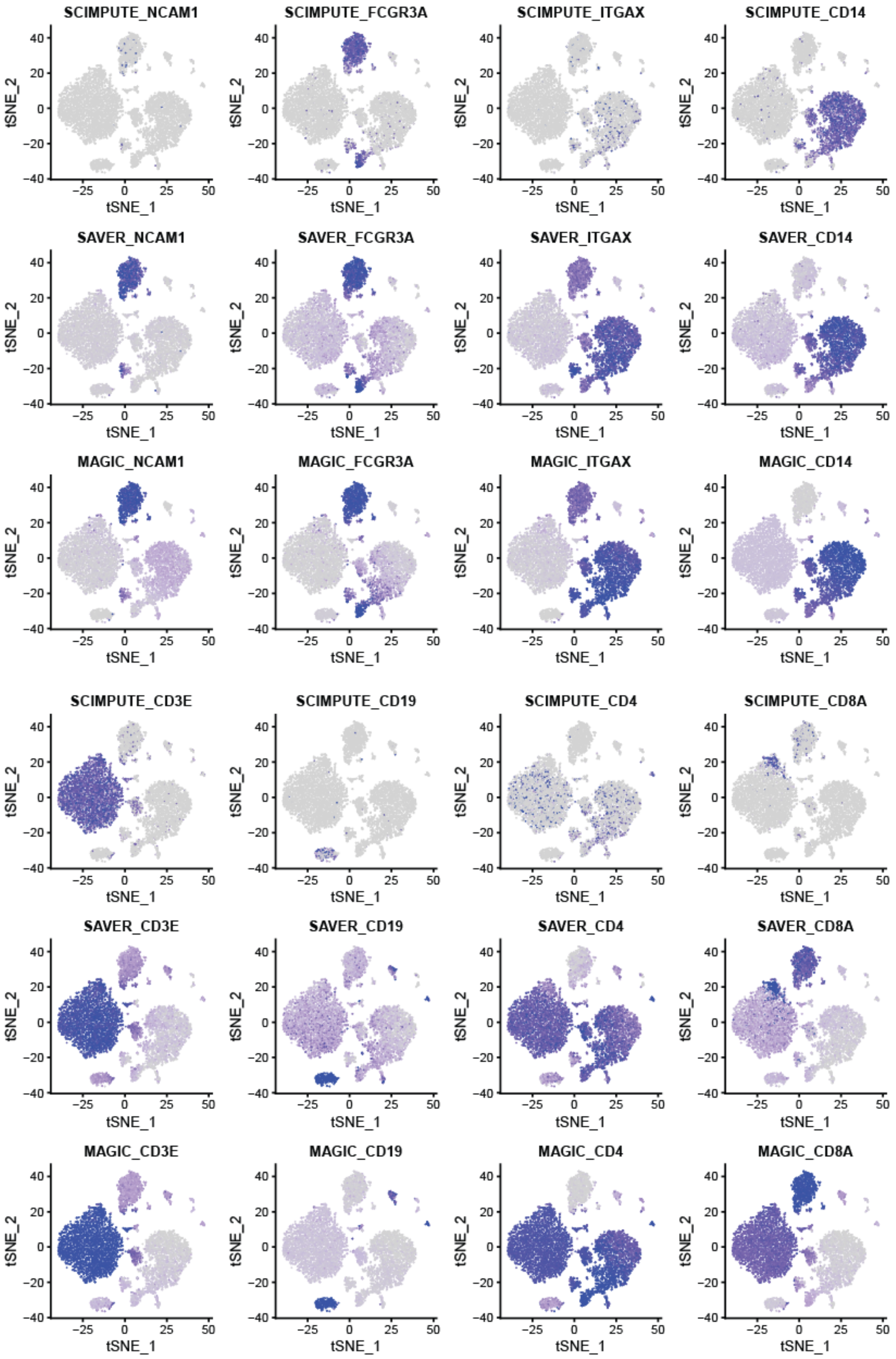

Supplementary Figure 5. tSNE visualizations colored by denoised RNA expression levels for other denoising methods for all eight protein-RNA pairs.

Supplementary Figure 6

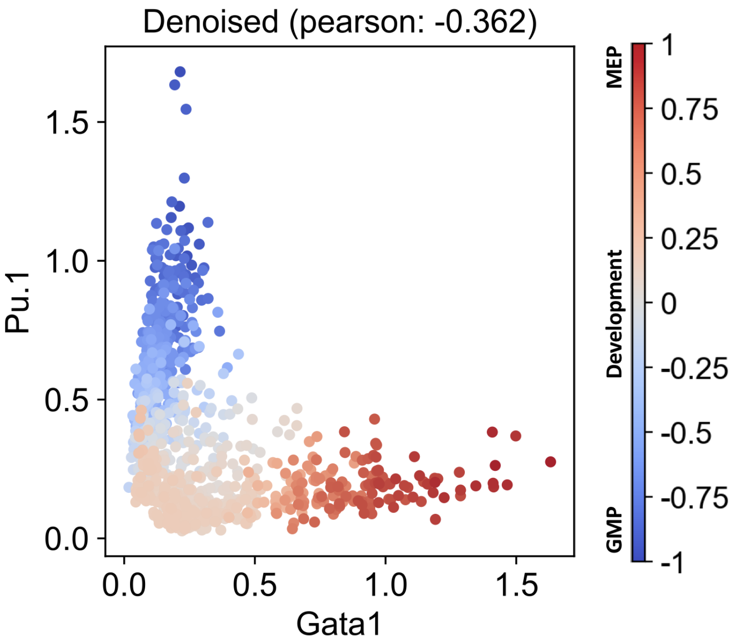

Supplementary Figure 6. X and Y axes depict *Gata1* and *Pu.1* expression levels across 939 cells with zero counts in original expression data, respectively.

Supplementary Table 1

Name	First Author	Summary
MAGIC	van Dijk	For imputation the original data matrix is right-multiplied to the Markov Affinity matrix from euclidean cell distances to describe the diffusion between cells.
SAVER	Huang	Calculates a posterior estimate from a Gamma-Poisson mixture model.
scImpute	Li	Clusters the data into K subpopulations. Calculates the dropout probability with a Gamma-Normal mixture model followed by a non-negative least squares regression model for genes with a high dropout probability.
DCA	Eraslan, Simon	Reconstructs expression using an autoencoder neural network with zero-inflated negative binomial likelihood loss function.

Supplementary Table 1. Overview of the four scRNA-seq denoising/imputation methods used in the comparison.
